# Bile Leak following Laparoscopic Cholecystectomy due to Perforated Duodenal Ulcer in Patient with Roux-en-Y Gastric Bypass

**DOI:** 10.1155/2021/6662433

**Published:** 2021-08-03

**Authors:** Amanda M. Marsh, Ayman Almousa, Thomas Genuit, David Forcione, Karin Blumofe

**Affiliations:** ^1^Department of General Surgery, Florida Atlantic University Schmidt College of Medicine, Boca Raton, Florida, USA; ^2^Boca Raton Regional Hospital, Baptist Health System, Boca Raton, Florida, USA

## Abstract

Perforated ulcers of the excluded stomach or duodenum are exceedingly rare in patients who have undergone Roux-en-Y gastric bypass surgery. The diagnosis of perforated ulcer after Roux-en-Y gastric bypass remains challenging as there is often absence of free air or contrast extravasation from the biliopancreatic limb. We present a patient with signs and symptoms of acute cholecystitis. Laparoscopic cholecystectomy was complicated by postoperative bile leak. EDGE procedure was performed to access the remnant stomach and endoscopic evaluation revealed a perforated ulcer in the posterior duodenal bulb. Although unusual, in patients with bariatric surgery and upper abdominal pain, differential diagnosis including perforated ulcer of the biliopancreatic limb must be considered and early surgical exploration is essential.

## 1. Introduction

Roux-en-Y gastric bypass (RYGB) is one of the most common bariatric procedures performed in morbidly obese patients worldwide. Due to the widespread use of proton pump inhibitors and the pharmacologic eradication of *Helicobater pylori* (*H. pylori*), the incidence of perforated peptic ulcer disease (PUD) has decreased in recent years [1]. This disease remains more prevalent in patients with known risk factors including the use of nonsteroidal anti-inflammatory drugs (NSAIDs), cigarette smoking, alcohol consumption, *H. pylori* infection, and hypersecretory states such as the Zollinger-Ellison syndrome [1]. Ulcer disease in the excluded segment after Roux-en-Y gastric bypass is a rare but potentially life-threatening complication with only 54 cases reported in the literature [2, 3]. The anatomical changes after RYGB lead to an atypical clinical presentation and can be a diagnostic challenge.

## 2. Case Reports

**Figure 1:**
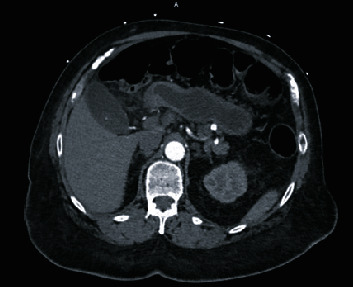
Preoperative CT scan shows mild distention of the gallbladder with gallstones and adjacent inflammatory stranding extending to the first and second portion of the duodenum.

A 67-year-old male patient presented to the emergency department at an academic-affiliated private hospital with one week of progressively worsening right upper quadrant (RUQ) abdominal pain that radiated to his back and right shoulder. The patient's past medical history was significant for hypertension, hyperlipidemia, non-insulin-dependent diabetes mellitus type II, and morbid obesity for which he had undergone a laparoscopic Roux-en-Y gastric bypass eight years prior with a resultant 150 lb. sustained weight loss. On evaluation, the patient was afebrile, normotensive, and with asymptomatic sinus bradycardia. A complete blood count and comprehensive metabolic panel were within normal limits, including WBC 8000/mcl without left shift. Physical examination revealed RUQ tenderness to palpation with a positive Murphy's sign and voluntary guarding. Abdominal ultrasound showed a dilated gallbladder with wall thickness of 3.3 mm and gallstones, without pericholecystic fluid or intra- or extra-hepatic biliary ductal dilation. Computed tomography (CT) of the abdomen with intravenous contrast showed a mildly distended gallbladder with gallstones, adjacent inflammatory changes extending to the first and second portions of the duodenum (D1 and D2) with a small amount of anterior perihepatic fluid, without additional free fluid or free air identified (Figure 1).

After careful consideration, the patient underwent laparoscopic cholecystectomy with intraoperative cholangiogram. The abdomen was entered under direct visualization and significant adhesions of the omentum and transverse colon were seen covering the gallbladder which required careful dissection for exposure. A bile-tinged exudate was seen covering the liver, gallbladder, and adjacent peritoneum which was concerning for possible gallbladder perforation. Once completely dissected free, the gallbladder was noted to be significantly distended without evidence of perforation or necrosis. A needle decompression was performed which returned normal green bile. The critical view of safety was obtained and transcystic cholangiogram was performed revealing normal intra- and extra-hepatic biliary anatomy without evidence of filling defects or bile leak. Next, cholecystectomy was performed in the standard fashion and the cystic duct was clipped and reinforced with a PDS endoloop. Due to the significant amount of inflammation, a Jackson-Pratt (JP) drain was placed in the gallbladder fossa and the patient tolerated the procedure well.

**Figure 2:**
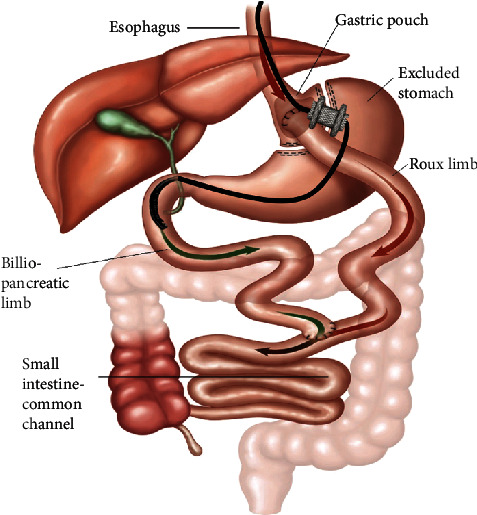
Depiction of Endoscopic Ultrasound-Directed Transgastric ERCP (EDGE procedure). Image provided by Dr. Prashant Kedia.

On postoperative day 1, over 500 cc of bilious drainage was noted from the JP drain consistent with a controlled bile leak. The patient was eventually advanced to a regular diet and treated with antibiotics, however, he continued to have persistent abdominal pain. His WBC increased to 12,000/mcl, but he remained afebrile with high output bile drainage of approximately 500 cc per day. On postoperative day 5, the patient underwent endoscopic retrograde cholangiopancreatography (ERCP) for further evaluation. Given the patient's RYGB anatomy, the gastric remnant was successfully accessed via endoscopic ultrasound-directed transgastric ERCP (EGDE procedure), with a 20 mm × 10 mm lumen-apposing metal Axios stent (Figure 2).

**Figure 3:**
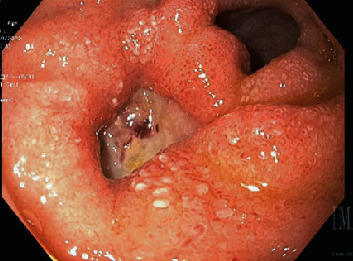
EDG shows a large fibrotic, chronic-appearing, penetrating duodenal bulb ulcer with fistulous tract in communication with the surgical drain.

ERCP demonstrated normal post-cholecystectomy anatomy without evidence of biliary leak and a normal pancreatic ductal system. The patient was treated prophylactically with sphincterotomy, biliary stent, and pancreatic stent placement. The gastrojejunal anastomosis was found to be widely patent and access to the biliopancreatic limb revealed a 2 cm deep, chronic-appearing, penetrating ulcer in the posterior duodenal bulb (Figure 3). The ulcer was biopsied at multiple sites and contrast injection at the ulcer base revealed a small fistula draining from the duodenum into the surgical JP drain, without evidence of free intraperitoneal contrast extravasation. Attempts were made to primarily close the ulcer with an endoscopic overstitch device (Apollo Endosurgery, Austin, TX) but failed due to significant ulcer-related fibrosis. Subsequently, endoscopic clips were used to partially reapproximate the ulcer edges by nearly 50%.

Over the next two days, with the addition of a twice daily proton pump inhibitor, the patient's abdominal pain improved significantly and the JP drain output decreased to less than 150 cc per day. The patient was able to tolerate an enteral diet and was discharged home with the JP drain in place. Histologic evaluation of the gallbladder revealed chronic cholecystitis with cholelithiasis and no evidence of malignancy. The duodenal ulcer pathology showed gastric antral-type mucosa with chronic inflammation, without evidence of *H. pylori* or malignancy. The JP drain was removed after two weeks and the patient felt significantly improved on follow-up examination. Repeat endoscopy at eight weeks confirmed the resolution of the duodenal ulcer and the Axios stent, biliary stent, and pancreatic stent were removed.

## 3. Discussion

We present a rare case of perforated duodenal ulcer after prior Roux-en-Y gastric bypass that presented as acute cholecystitis with postoperative bile leak. Even though the patient had radiologic and physical exam findings consistent with acute cholecystitis, we suspect the patient's ulcer perforated prior to his presentation and contributed to the significant inflammation and distention of the gallbladder. Due to the posterior location of D1 and the adhesions in the RUQ, duodenal ulcer or perforation was not appreciated at the time of the initial operation. For suspected bile leak, ERCP is a safe and effective treatment option post-cholecystectomy, as was performed during this case [4, 5].

While the incidence of PUD after RYGB is not known, a recent review of the literature by Plitzko et al. summarized a total of 54 patients from 5 case series and 18 case reports [3]. Of these reported cases, 28% of patients presented with gastrointestinal bleeding and 70% of patients presented with perforated ulcers [3]. The site of bleeding occurred 53% in the remnant stomach and 47% in the remnant duodenum, whereas perforation was localized to the remnant stomach in 34% of cases and in the remnant duodenum 66% of cases [3]. The leading symptom at presentation was epigastric or upper abdominal pain, although bleeding, melena, weakness, and anemia were also common findings [3].

Several mechanisms have been proposed to explain the pathophysiology of PUD in the biliopancreatic limb following bariatric surgery. *H. pylori* has been clearly implicated in the formation of ulcers in the gastric bypass population by weakening the mucosal protective barriers [2]. Mucosal injury may also result from the ingestion of NSAIDs or alcohol consumption [2]. After RYGB, there is continued but diminished acid excretion in the excluded stomach [3]. Gastrin levels are decreased, yet gastric mucosa maintains the ability to respond to vagal and hormonal stimuli, thus preserving an acidic environment [2, 3]. Furthermore, bile refluxing into the gastric remnant also contributes to mucosal injury as there is a delay in the release of pancreatic bicarbonate secretion, which typically serves as a buffer for gastric acid secretion [2, 3].

Perforated ulcers typically do not result in pneumoperitoneum or free fluid from the excluded segments [3]. Typical CT scan findings may show duodenal wall thickening, fat stranding, or periduodenal fluid [1]. However, negative radiologic findings do not exclude a perforated viscus and must be met with a lower threshold for surgical exploration [2, 3]. While duodenal ulcer formation in the excluded segment after RYGB is extremely rare, rapid weight loss and the development of gallstones is a well-known complication of bariatric surgery [6–8]. Therefore, in patients with a history of gastric bypass who present with findings consistent with cholelithiasis or cholecystitis, the diagnosis of duodenal ulcer or perforation may be overlooked.

The anatomy after RYGB impedes routine access to the remnant stomach and duodenum but there are various ways to access the excluded segments including open, laparoscopic, or endoscopic ultrasound-guided access [3, 7]. Upper endoscopy is a minimally invasive approach that is considered a first-line approach for the diagnosis and treatment of upper gastrointestinal bleeding, anastomotic leak, or remnant gastritis in these patients [1, 3]. Options to address bleeding or perforated ulcers after RYGB include oversewing with omental patch, remnant gastrectomy, or percutaneous transgastric endoscopy [3, 9]. In this case, we successfully accessed the remnant segment via EDGE procedure. EDGE procedure has been shown to have comparable technical and clinical success rates when compared to laparoscopic-assisted ERCP, with the use of lumen-apposing metal stents, endoscopic sutures, and over-the-scope clips [6]. Despite evolving technology and treatment outcomes, further investigation is required to prevent ulcer disease in the RYGB patient population.

In conclusion, clinicians must maintain a high index of suspicion in patients with history of Roux-en-Y gastric bypass who present with acute abdominal pain and ulcer disease of the excluded stomach or duodenum must be considered. The use of ultrasound-guided transgastric ERCP has been suggested as a safe and highly effective approach for patients with gastric bypass anatomy.

## Data Availability

The data used to support the findings of this study are included within the article.
